# The Role of Injectables in the Treatment and Prevention of Cancer-Associated Thrombosis

**DOI:** 10.3390/cancers15184640

**Published:** 2023-09-20

**Authors:** Mariasanta Napolitano, Sergio Siragusa

**Affiliations:** Haematology Unit, Thrombosis and Haemostasis Reference Regional Center, University of Palermo, 90121 Palermo, Italy; sergio.siragusa@unipa.it

**Keywords:** cancer-associated thrombosis, venous thromboembolism, injectables, unfractionated heparin, low-molecular weight heparins, tinzaparin

## Abstract

**Simple Summary:**

People with cancer are at an increased risk of developing blood clots. Both cancer and anti-cancer treatment are responsible for this risk. In some types of cancer, such risk is higher than in others. There are different types of blood thinners also known as anticoagulants. Blood thinners prevent the recurrence of deep venous thrombosis (a blood clot in the limbs) or pulmonary embolism (a blood clot in the lungs) in people with cancer. In some clinical situations and, specifically, in patients with active cancer, injectable blood thinners are safer than tablets to be taken orally and work just as well.

**Abstract:**

Cancer-associated thrombosis (CAT) is a leading cause of death among patients with cancer. CAT can manifest itself as venous thromboembolism (VTE), in the form of deep vein thrombosis or pulmonary embolism, or arterial thromboembolism. The pathophysiology of CAT is complex and depends on cancer-, patient-, treatment- and biomarkers-related factors. Treatment of VTE in patients with cancer is complex and includes three major classes of anticoagulant agents: heparin and its derivatives, e.g., low molecular weight heparins, direct oral anticoagulants (DOACs), and vitamin K inhibitors. Given the tremendous heterogeneity of clinical situations in patients with cancer and the challenges of CAT, there is no single universal treatment option for patients suffering from or at risk of CAT. Initial studies suggested that patients seemed to prefer an anticoagulant that would not interfere with their cancer treatment, suggesting the primacy of cancer over VTE, and favoring efficacy and safety over convenience of route of administration. Recent studies show that when the efficacy and safety aspects are similar, patients prefer the oral route of administration. Despite this, injectables are a valid option for many patients with cancer.

## 1. Introduction

Cancer-associated thrombosis (CAT) is a common cardiovascular complication that manifests itself in individuals with a malignancy. CAT may be the first sign prompting the diagnostic workup that leads to the discovery of occult cancer, or more typically, occurs within six months of cancer diagnosis [1,2,3].

The clinical presentation of CAT includes venous thromboembolism (VTE) and arterial thromboembolism (ATE). VTE is the most frequent clinical presentation of CAT and includes both deep vein thrombosis (DVT) and pulmonary embolism (PE). However, VTE is not limited to DVT and PE and may involve atypical sites such as the upper extremities, splanchnic and cerebral veins. ATE, instead, primarily manifests itself as myocardial infarction, cerebrovascular event, or peripheral artery disease. Importantly, CAT is the second leading cause of death after cancer itself in patients with cancer who have a 47 times higher annualized rate of death from VTE than the general population [4,5].

CAT has profound consequences for the patient (symptoms and mortality, worsened overall survival, worsened quality of life, delay or interruption of anti-cancer treatment due to occurrence of CAT, risk of recurrence, risk of bleeding when on anticoagulant treatment) and for the healthcare systems (the costs of emergency care visits or hospitalization, or anticoagulant treatment) [6]. Effective treatment and/or prevention of CAT is thus paramount.

Several therapeutic options are available for the management of CAT, such as unfractionated heparins (UFHs), low molecular weight heparins (LMWHs), fondaparinux, and direct oral anticoagulants (DOACs) [7,8,9,10,11,12,13]. Injectables constitute a versatile choice to manage patients with cancer during the active phase of the disease (i.e., in individuals who: (i) received a cancer diagnosis within the previous 6 months; (ii) have recurrent, regionally advanced or metastatic disease; (iii) received cancer treatment within the previous 6 months; or (iv) have a hematological neoplasm and did not obtain complete remission), and are not associated with significant safety issues) [14]. Instead, guidelines warn that the administration of DOACs may put at risk patients with inoperable gastrointestinal (GI), GI comorbidities or toxicity, genitourinary cancer, severe renal dysfunction (i.e., creatinine clearance (CrCL) >15–<30 mL/min), and thrombocytopenia [7]. The primary aim of this review is to discuss the role of injectables in the treatment of CAT. Furthermore, we highlight the most challenging scenarios for which the treatment with LMWH is recommended.

## 2. Epidemiology of Cancer-Associated Thrombosis

The introduction of targeted therapies together with the increased survival of patients with cancer has broadened the population of patients with cancer at risk of developing CAT [6]. Amongst patients with cancer, the prevalence of VTE is much higher than in the general population. It is estimated that persons diagnosed with cancer have an average risk of developing VTE ~12 times higher than the general population, which can become ~23 times higher when the patient is exposed to chemotherapy [15,16]. A recent Austrian nationwide analysis involving about 160,000 individuals with cancer and more than 8 million people without cancer showed an over 10-fold difference (4.6% versus 0.4%) in the prevalence of VTE [17]. An increase from 1% in 1997 to 3.4% in 2017 in the 12-month incidence of VTE was observed amongst Danish patients with cancer [16]. According to estimates, approximately 4–20% of patients with cancer will experience VTE at some stage of their disease [18]. The recent progress in cancer therapy even if improving survival outcomes, has not managed yet to limit the risk of developing VTE in patients with cancer.

## 3. Pathogenesis

The pathophysiology underlying the development of VTE events, regardless of being or not associated with cancer, consists of one or more elements of the triad named after Rudolf Virchow and composed of hypercoagulability, venous stasis, and endothelial damage [19]. Patients with cancer often display abnormalities in each element of Virchow’s triad [18]. Hypercoagulability refers to a condition that facilitates thrombus formation and involves several pathways, often coactivated, such as direct activation of coagulation, inhibition of fibrinolysis, cancer-induced platelet aggregation, and inflammation.

Direct activation of coagulation may be secondary to the presence in the circulation of cytokines secreted directly by tumor cells and their interaction with the tumor microenvironment, such as tissue factor (TF) and, membrane microparticles (MPs) bearing TF, cancer procoagulant (CP) and heparinases. These molecules not only shift the hemostatic balance in a pro-coagulant sense but are probably also involved in mechanisms of tumor diffusion. Moreover, molecules responsible for the inhibition of the fibrinolytic system, such as the plasminogen activator inhibitors 1 and 2 (PAI1, PAI2) are also overexpressed on the surface of the neoplastic cells, which further destabilizes the hemostatic balance [20].

In patients with cancer, platelets are activated by ADP, thrombin, some metalloproteases, and interleukin (IL)-6 produced by tumor cells that induce the expression of adhesion molecules on platelet surface (e.g., P-selectin) increasing their tendency to aggregation [21]. Platelets contribute to thrombin generation, which may, in turn, trigger a vicious circle promoted by the protease-activated receptors (PARs)/tumor growth factor (TGF)-beta signaling [22].

The role of inflammation in CAT cannot be underestimated. Tumor cells interact with the tumor microenvironment and with cells of the immune system and increase, both locally and systemically, the levels of acute phase proteins and inflammatory mediators. This, in turn, increases the release of TF, von Willebrand factor, PAI-1, vascular endothelial growth factor (VEGF), and a concomitant reduction in protein C levels that together lead to a pro-coagulant state [23,24]. Furthermore, leukocyte activation induced by their direct interaction with the tumor mass causes an increase in pro-thrombotic activity through the expression of specific adhesion molecules on the lymphocyte surface and through the formation of neutrophil extracellular traps (NETs), an intricate network of externalized DNA, histones, and proteases. NETs facilitate aggregation and thrombus formation [25], as well as participate in the processes of immune escape of the tumor and metastasis [26]. Moreover, pro-inflammatory mediators, such as IL-1b, IL-6, tumor necrosis factor alpha (TNF-a), and lipopolysaccharides, together with pro-angiogenic factors (VEGF and fibroblast growth factor (FGF)), make the vascular surface more prone to platelet adhesion [18].

Surgical interventions cause several structural and humoral modifications promoting the state of hypercoagulability. The origin of these alterations is multifactorial, including an overexpression by the tumor tissue of procoagulant substances, endothelial damage, an increase in platelet activation, and an increased expression of TF by monocytes [27,28].

Supportive medical therapy can also induce pro-coagulant changes. In particular, the use of erythropoiesis-stimulating agents, transfusions of packed red blood cells, transfusions of platelet concentrates, and granulocyte-stimulating growth factors (G-CSF) correlates with an increased rate of VTE development [28]. Last but not least, venous stasis caused by the compression of blood vessels by tumors or prolonged immobility in critically ill patients with cancer contributes to the pathophysiology of CAT [29].

Risk factors and biomarkers for the development of CAT are tumor-, patient- and therapy-related and have been extensively reviewed elsewhere (e.g., [28,30]).

## 4. Risk Assessment Models in the Prevention of Cancer-Associated Thrombosis

As the risk for VTE in cancer is multifactorial, no single biomarker can be used to predict it. Over the years, several risk assessment models (RAMs) have been developed to identify patients with cancer at increased risk of VTE.

The Khorana score, currently recommended by guidelines, was the first risk prediction model for VTE in ambulatory patients with cancer [31]. It evaluates five parameters (type of cancer, three components of the complete blood counts, and body mass index (BMI)) prior to chemotherapy. Each variable is assigned 1 point, whilst the presence of a tumor type from the very high-risk category (i.e., pancreatic and gastric cancers) scores 2 points. The score was derived from data on 2701 patients and subsequently validated in 35,000 patients [31]. Since its validation, several variations of the original Khorana score have been proposed and include Vienna, PROTECHT, ONKOTEV, COMPASS-CAT, Tic-ONCO, IMPEDE, or SAVED scores. These modified RAMs add additional or remove parameters (e.g., add D-dimer and soluble P-selectin in the Vienna score, remove MBI/add a type of chemotherapy in the PROTECHT score, or add genetic risk in the Tic-ONCO score) and can be tumor type specific (e.g., IMPEDE and SAVED can only be applied to patients with multiple myeloma) [32].

**Figure 1:**
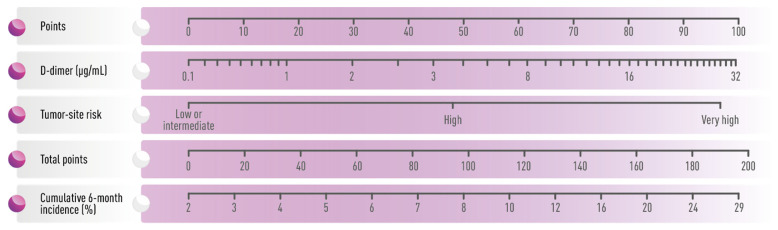
Nomogram for the prediction of the 6-month risk of venous thromboembolism according to Pabinger et al., 2018. Adapted from [33].

Recently, a simple two-variable model has been proposed by Pabinger et al. [33]. It incorporates one clinical parameter (i.e., tumor site category stratified into “low/intermediate”, “high”, and “very high” risk) and one biomarker (i.e., D-dimer) (Figure 1) and predicts the risk of VTE in ambulatory patients with solid cancer. This score has been validated internally and externally and seems to possess a better predictive value for VTE than the original Khorana score [33]. At present, there are no validated risk tools to predict ATE in patients with cancer.

## 5. The Impact of Cancer Type, Stage, and Treatment

Studies have shown that patients with some cancer types are at an increased risk of VTE compared to other types. These are pancreas, brain, stomach, kidney, uterus, bladder, lung, colon, and hematological malignancies, whereas such risk is lower in breast and prostate cancer [34,35,36]. The histological type of the tumor seems also to play a role in the development of VTE. Patients with lung adenocarcinoma are at a higher risk of developing VTE than patients with squamous cell carcinoma of the lung, whereas, in the setting of ovarian cancer, mucin-producing adenocarcinomas are linked to a higher risk of developing DVT and PE than those that do not produce mucins [37,38]. The risk of developing VTE is the highest in the first three months since the diagnosis of cancer and metastatic disease is associated with an increased risk of VTE [36]. Metastases to bones, liver, lung, and adrenal glands, as well as those to regional and distal lymph nodes, result in increased hypercoagulability and, thus, increased risk of VTE; tumor grade may also influence the risk of VTE [39,40,41,42]. The risk of developing VTE in patients with grade 3 and grade 4 disease is twice that of patients with low-grade disease (HR 2.0; 95% CI, 1.1–3.5) [39].

All therapeutic modalities used to treat cancer are associated with the risk of developing VTE, albeit through diverse mechanisms, some of which are still not fully understood [43]. In the Danish cohort, treatment modalities resulted in important risk factors with the subdistribution HRs of 3.4 (95% CI, 3.1–3.7) for chemotherapy, 4.1 (95% CI, 3.4–4.9) for protein kinase inhibitors, 4.4 (95% CI, 3.8–5.2) for antiangiogenic therapy, and 3.6 (95%CI, 2.8–4.6) for immunotherapy [16]. Major surgery is linked to a high risk of VTE that remains high for 30 days afterward with the incidence of VTE in the post-operative period reaching 2% [44]. Similarly, surgery for long bone metastases including intramedullary nails, endoprosthetic reconstruction, or place-and-screw fixation, results in a high 90-day risk of VTE, with a 90-day incidence of 6% (44/682) [45]. VTE is more frequent following soft tissue (10.6%) or lung (8.1%) surgery [46]. Chemotherapy, due to direct vascular toxicity or endothelial cell destruction caused by platinum-based regimens, is a significant risk factor for VTE. Studies suggest a six-fold increase in VTE when in treatment with chemotherapy, however, not all chemotherapeutic agents increase the risk of VTE to the same degree [47]. About 18% of patients with cancer developed VTE after receiving cisplatin or within 4 weeks after the last dose of chemotherapy [48]. Hormonal therapy, such as tamoxifen, in patients with breast cancer, increases the 5-year risk of developing VTE from 0.5% to 1.2% [49]. Such risk increases further if tamoxifen is combined with chemotherapy (from 1.4% for tamoxifen alone to 10.8% for the combination) [50]. Immunotherapy and molecularly targeted anti-cancer therapies, such as tyrosine kinase inhibitors and antiangiogenic agents, are changing the overall outcomes of anticancer therapies but require more studies to fully understand their relationship with the development of VTE [28,47]. Two metanalyses showed that the use of drugs that inhibit epidermal growth factor receptors, e.g., cetuximab and panitumumab, is associated with an increased risk of VTE (relative risk (RR) 1.32 and 1.46, respectively) [51,52]. In addition, immunotherapy seems to increase the risk of VTE, with a study showing that the risk of developing VTE under immunotherapy and chemotherapy is similar [53].

## 6. Treatment and Prophylaxis

Treatment of VTE in patients with cancer is complex and includes three major classes of anticoagulant agents: LMWHs, DOACs, and vitamin K inhibitors (see Table 1 for their major characteristics). According to the latest guidelines, LMWHs and DOACs are preferred over vitamin K antagonists for the initial treatment of established VTEs with an initial duration of three to six months [7,9,10,11,12,54,55]. The available guidelines on prophylaxis and management of VTE do not provide differentiated recommendations for specific types of cancer. Supplementary Table S1 summarizes the clinical trials that provided the evidence for the recommended approaches.

In individuals in whom LMWHs are absolutely contraindicated and for those who relapse on full-dose anticoagulation, an inferior vena cava filter is recommended for the initial treatment [10].

Considering that the thrombotic risk caused by cancer is a constant, all guidelines prefer a long-term anticoagulation strategy over treatment lasting less than or equal to 6 months [10,11,12,54,55]. Despite this, clinical trials conducted to date provide no data on follow-ups longer than 12 months of treatment. Clearly, the risk of bleeding deriving from taking anticoagulant therapy indefinitely must be carefully weighed to avoid the risk of major/non-major clinically relevant bleeding.

**Figure 2:**
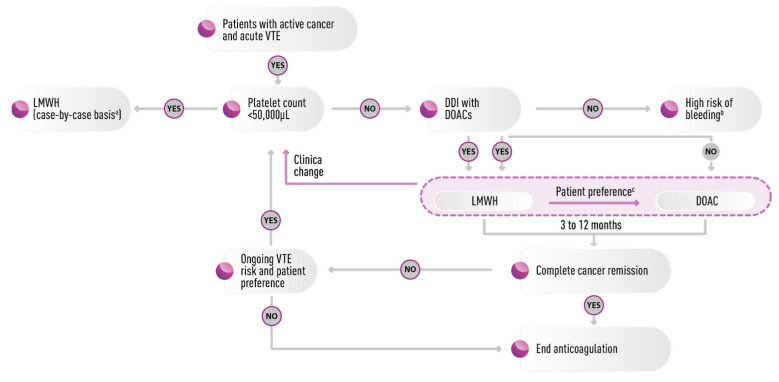
Potential approach to the treatment of venous thromboembolism in cancer patients. ^a^ Reduced- or full-dose after a transfusion. ^b^ Includes patients with cancer of gastrointestinal tract and cancer-unrelated risk factors. ^c^ Decided on a case-by-case basis weighing up relative risks and benefits. DDI, drug–drug interactions; DOAC, direct oral anticoagulant; LMWH, low-molecular-weight heparin; VTE, venous thromboembolism. Adapted from [59].

The primary choice of extended treatment is LMWHs or DOACs. Although from the point of view of efficacy, there are no substantial differences, DOACs have a proven increased risk of major bleeding and a greater number of drug interactions. The choice of the type of treatment should therefore always take into consideration the clinical context, the characteristics of the patient, their individual risk of thromboembolic recurrence or major bleeding, and patients’ preferences (Figure 2). Early studies suggested patients’ preference for effective and safe treatments and those that would not interfere with their cancer treatment. The most recent studies show that patients prefer the oral route of anticoagulation, with efficacy and safety being similar to injectables [56,57,58].

## 7. Special Clinical Settings

Several factors may challenge the successful treatment of VTE in patients with cancer. Drug–drug interactions altering the efficacy of chemotherapy or anticoagulation, and the occurrence of adverse events in the form of thrombocytopenia, major bleedings, or clinically relevant non-major bleedings, are the most frequent risks associated with anticoagulant treatment. Therefore, a physician should consider the risk of bleeding, the patient’s age, type of cancer, disease stage, and anti-cancer treatment regimen when prescribing a VTE prophylaxis schedule.

In patients with mild renal insufficiency, a slight increase in BMI, or mild obesity, anticoagulants for the prevention or treatment of VTE do not need to be specifically adjusted. Instead, other patient categories warrant special care. These include individuals with severe renal impairment, severe and moderate obesity, and those who underwent proximal GI surgery affecting oral drug absorption. Injectable anticoagulants are largely preferred in patients with potentially impaired GI absorption [13] and in those with moderate to severe thrombocytopenia [61].

### 7.1. Drug–Drug Interaction

Many chemotherapeutics inhibit the activity of enzymes involved in drug metabolisms such as P-glycoprotein or cytochrome CYP3A4, otherwise involved in the metabolism of DOACs, meaning that drug–drug interactions may be more common in patients with cancer than in the general population [62,63]. In addition, drug–drug interactions may impact both cancer and VTE treatment effectiveness and safety [64]. Accordingly, consensus guidelines suggest that the use of DOACs should be avoided in patients at high risk of drug–drug interactions [8,9,10,14].

### 7.2. Cerebral Involvement

Compared to other types of cancer, patients with primary brain tumors and brain metastases are at an increased risk of developing CAT. VTE occurs in up to 30% of patients with glioma and in up to 20% of those who have brain metastases or who develop central nervous system lymphomas [65]. The reason behind such high rates of VTE in patients with brain tumors has been linked to plasmin inhibition, thromboplastin release, and increased procoagulant and platelet aggregation activity [66]. Prophylactic and even therapeutic anticoagulation seemingly did not increase the risk of major intracranial bleeding in patients with brain cancer, albeit there is no evidence to support long-term primary pharmacological VTE prophylaxis in this group of patients [66]. Moreover, in the case of surgery, brain tumors and tumor metastases carry a high risk of perioperative VTE [66]. The rate of postoperative VTE after craniotomy to surgically treat brain tumors versus neurosurgical craniotomies was two times higher (3.2% versus 1.4%) [67]. Also, surgery for brain metastases is associated with a higher risk of VTE than surgery for primary cerebral tumors (19% versus 7.5%) [68]. Given such a high prevalence of VTE is linked to brain cancer surgery, VTE prophylaxis is necessary in this setting, but it must be carefully balanced with the risk of intracranial bleeding. Studies and guidelines recommend the use of pharmacological and mechanical prophylaxis in these patients [66]. In the past, patients with brain tumors were treated with the placement of vena cava filters; however, good quality evidence on this issue is lacking [9,10]. Newly emerging findings hint that DOACs may be a more efficient and safer choice than other anticoagulants in this setting. All challenges are described in a recent review article [69].

### 7.3. Low and Unstable Platelet Count

Coexisting VTE and thrombocytopenia are frequent in patients with active cancer. In a retrospective cohort at Beth Israel Deaconess Medical Center, 3635 unique CAT events were identified over 10 years. About 1 in 4 episodes of VTE occurred in the presence of thrombocytopenia, and ~1 in 10 of those patients had platelet counts < 50,000/µL. [70]. In that cohort, thrombocytopenia (platelets < 100,000/µL) occurred in 22% (95% CI, 21–24%) of patients with CAT and solid tumors and in 47% (95% CI, 43–51%) of patients with CAT and hematologic cancers. Severe thrombocytopenia (platelet < 50,000/µL) occurred in 7% (95% CI, 6–8%) and 30% (95% CI, 27–34%) of patients with solid/hematologic cancers, respectively [71]. In the case of hematologic malignancies, thrombocytopenia can be secondary to bone marrow infiltration by the leukemic blasts at disease onset or relapse or due to myelotoxic effects of antileukemia therapy [61]. Acute and chronic leukemias, myelodysplastic syndromes, and aplastic anemia may lead to severe thrombocytopenia since diagnosis. Thrombocytopenia may thus last several weeks or months, increasing the risk of bleeding. Thrombocytopenia due to anticancer therapy occurs via various mechanisms that can be divided into two groups: bone marrow suppression and effects on circulating platelets [72]. Alkylating agents, cyclophosphamide, cisplatin, and proteasome inhibitors inhibit pluripotent stem cells, act on later megakaryocyte progenitors, and decrease platelet shedding by megakaryocytes. Immune checkpoint inhibitors, oxaliplatin and fludarabine, cause immune thrombocytopenia. Gemcitabine and mitomycin C cause thrombotic microangiopathy, while venetoclax and cisplatin increase platelet apoptosis. Kinase inhibitors inhibit platelet kinase activity [72]. Thrombocytopenia does not protect from VTE but significantly increases the risk of bleeding, mostly when using anticoagulants [70]. In patients with cancer and thrombocytopenia, the risks and benefits of anticoagulation must be carefully balanced. Due to the short half-life, the ease of modifying dosing, and minimal drug–drug interactions, UFH and LMWH are the most commonly administered anticoagulants in the setting of CAT and thrombocytopenia [70]. In addition, LMWHs are recommended at a dose adjusted to platelet count in patients at high risk of bleeding [61].

### 7.4. Frail Patients

Frailty is usually defined as a multidimensional syndrome involving the loss of energy, physical ability, cognition, and health and is characterized by an excessive vulnerability of the individual to endogenous and exogenous stressors [73,74]. Old age is one of the major determinants of frailty in patients with CAT [74]. Frail patients with CAT require special care. Anticoagulant treatment in this population at high risk of VTE and bleeding is particularly complex due to age, comorbidities (e.g., chronic kidney disease), polypharmacotherapy with potential drug–drug interactions, anemia, thrombocytopenia, mobility, nutritional status, risk of falls, and reduced life expectancy. Treatment adherence in patients with cognitive impairment may also be diminished [74]. Noteworthy, SELECT-D, Hokusai VTE cancer, and ADAM-VTE trials enrolled patients at least ambulatory and capable of self-care (Eastern Cooperative Oncology Group performance status of ≤2). For this reason, the results of these studies may not be applicable to frail patients [75]. The real-world data from the Registro Informatizado Enfermedad TromboEmbolica (RIETE) registry showed that 42% of patients with VTE were frail (age ≥ 75 years, CrCl ≤ 50 mL/min, and/or body weight ≤ 50 kg). This registry collected data on all patients with VTE; ~20% of them have cancer. The subgroup analysis of the RIETE registry showed that during anticoagulation, patients in the frail subgroup had fewer VTE recurrences and more severe bleeding events than those in the non-fragile subgroup [76]. There is no specific guidance for anticoagulation in frail patients. The general guidelines should be followed and tailored as needed.

### 7.5. Renal Insufficiency

Chronic renal failure is frequently encountered in patients with cancer. In fact, between 52.9% and 87% of patients with cancer have a glomerular filtration rate (eGFR) value < 90 mL/min/1.73 m^2^, while eGRF values < 60 mL/min/1.73 m^2^ were observed in 11.8–25% of individuals with cancer [77]. From the perspective of anticoagulant therapy, an impairment in renal function leads to an increased risk of bleeding, especially major bleeding, when anticoagulant plasma levels increase. This is particularly true for DOACs as an important percentage of these drugs is removed through the renal elimination pathway. Their use must thus be evaluated with caution for filtration rate values < 30 mL/min/1.73 m^2^. In pivotal RCTs, patients with severe renal insufficiency (CrCl < 30 mL/min) were excluded [78]. Moreover, there are no literature data to support the use of DOACs at eGRF levels < 15 mL/min/1.73 m^2^. An observational study that analyzed the effects of anticoagulant therapy with VKAs or LMWHs in patients with cancer and renal insufficiency showed that patients with stage 4 and 5 renal failure were at the greatest risk of major bleeding when under treatment with LMWHs, advising caution in the use of these molecules as well [79]. Guidelines recommend dose adjustment of LMWHs guided by monitoring the anti-Xa level [7]. Given its high molecular weight, tinzaparin has the lowest potential to accumulate, whereas enoxaparin has the highest [80]. For tinzaparin, no dose reductions are necessary up to CrCl values of 20 mL/min [81].

### 7.6. Liver Impairment

Functional impairment of liver function may lead to a reduced ability to metabolize DOACs. All DOACs are metabolized by cytochrome P450 (see Section 7.1). When treated with DOACs, patients affected by liver disease are at a higher risk of bleeding than those with normal liver function due to drug accumulation. Similarly to patients with severe kidney impairment, individuals with liver disease were excluded from pivotal RCTs (an exclusion criterion based on levels of ALT/AST) [82]. None of the DOACs is recommended for use in patients in Child–Pugh class C [83]. It is advisable to treat patients with impaired hepatic function due to liver involvement by cancer and CAT with LMWHs [82].

## 8. Injectables

In contrast to the newer classes of DOACs, the injectable anticoagulants are administered intravenously or subcutaneously. They include UFH, dalteparin, enoxaparin, tinzaparin, and fondaparinux (Table 2). Dalteparin, enoxaparin, and tinzaparin are classified as LMWHs, and fondaparinux is a synthetic heparin pentasaccharide. Argatroban and bivalirudin are injectable direct thrombin inhibitors that are used in patients with heparin-induced thrombocytopenia (HIT) and HIT with thrombosis, rare complications of treatment with UFH, or more rarely with LMWHs, and will not be discussed in this review [84,85]. The injectable anticoagulants are European Medicines Agency (EMA)- and Food and Drug Administration (FDA)-approved for prophylaxis and/or treatment of VTE at the doses specified in Table 3. Please note that these are doses recommended by ESMO. Local guidelines should be followed when administering treatment to patients with cancer and CAT. In a single-center analysis, switching between the molecules within the class of LMWHs occurred in ~15% of patients and was as frequent as the class switch [86].

### 8.1. Unfractionated Heparin

Historically, UFH was the standard treatment for the treatment and prophylaxis of VTE. UFH is a mixture of glycosaminoglycans that bind to antithrombin III (AT-III) via a unique pentasaccharide sequence. Such binding enhances antithrombin’s inhibition mainly of factor Xa and factor IIa (thrombin) coagulation factors. UFH also binds cells and other plasma proteins. This ubiquitous binding property is responsible for the unpredictable pharmacodynamics and pharmacokinetics of UFH that can lead to heparin-induced thrombocytopenia or osteoporosis [88]. UFH must be given parenterally, usually by slow intravenous infusion, and requires frequent monitoring through the measurement of the partial prothrombin time [89].

A systematic review of the evidence for the relative efficacy and safety of LMWHs and UFH for perioperative thromboprophylaxis in patients with cancer was performed by Matar et al. The authors included 20 randomized clinical trials (RCTs; 9771 patients) that used preoperative prophylactic anticoagulation with UFH or LMWHs. The meta-analysis did not exclude conclusively a beneficial or harmful effect of LMWHs versus UFH on mortality and embolic outcomes in patients with cancer [90].

The treatment with UFH was compared to LMWH or fondaparinux to obtain pharmacologic prophylaxis of VTE in critically ill patients with cancer. The authors assessed whether LMWH was more effective than UFH at reducing in-hospital rates of VTE and improving clinical outcomes. The study included 103,798 patients from the Premier Database; 75,321 (72.6%) of them received LMWH and 28,477 (27.4%) UFH. The results showed that prophylaxis with LMWH did not decrease the incidence of VTE (5.32% versus 5.50%) but reduced the frequency of PE (0.70% versus 0.99%), significant bleeding (defined as discharge diagnosis of bleeding absent at admission and/or administration of two or more units of packed red blood cell in 24 h; 13.3% versus 14.8%) and HIT (0.06% versus 0.19%) [91].

In patients with cancer, a non-bridging therapy with UFH may be useful to reduce the risk of VTE before invasive procedures for which DOAC interruption was performed. In a study, 68 patients were treated with UFH and 74 were not. The rates of VTE recurrence (6% versus 1%, RR: 4.4, 95% CI, 0.50–38.0, *p* = 0.19), non-major bleeding (4% versus 3%, RR: 1.6, 95% CI, 0.28–9.48, *p* = 0.67), as well as major bleeding (0% versus 4%, RR—not estimated, *p* = 0.25), respectively, in the bridging and non-bridging with UFH groups, confirmed the possible usefulness of bridging with UFH [92].

In patients with brain tumors undergoing neurosurgery, VTE prophylaxis with UFH was found to be safer than other interventions (DOACs, LMWHs, or intermittent pneumatic compression) as far as the occurrence of major and minor bleeding, and all-cause mortality were concerned. DOACs, however, were more effective in VTE prophylaxis in a recent systematic review and Bayesian network meta-analysis of 10 RCTs that included 1128 patients with brain cancer who had neurosurgery [93].

### 8.2. Low molecular Weight Heparins

LMWHs are a combination of heparin molecules with a considerably lower molecular weight than UFH. Compared to UFH, LMWHs have greater bioavailability, longer half-lives, more predictable dose–response, and safety. LMWHs are obtained through depolymerization of heparin. They are administered subcutaneously and in the majority of patients, no monitoring is necessary. LMWHs inhibit coagulation by activating antithrombin, which binds to and inhibits factor Xa. The inactivation of Xa results in prothrombin not being activated to thrombin, thereby not converting fibrinogen into fibrin for clot formation [89].

Numerous trials have compared LMWHs to VKAs in the treatment of the acute phase of thrombotic complications. In the CLOT trial, 676 patients were randomized to receive dalteparin or warfarin for a duration of 6 months of treatment [94]. A meta-analysis conducted by the Cochrane Collaboration confirmed the advantage of LMWHs over VKAs in reducing VTE recurrence (RR 0.58; 95% CI, 0.43–0.77), while there were no differences in terms of major bleeding and mortality [95]. A study showed that long-term (up to 2 years) LMWH in patients with residual vein thrombosis was effective and safe. Moreover, it was well tolerated and did not negatively impact the quality of life of the patients in the treatment [96].

Amongst LMWHs, dalteparin and tinzaparin, and to a lesser extent, enoxaparin, are by far the most studied LMWH in the setting of CAT.

#### 8.2.1. Dalteparin

Dalteparin sodium is an LMWH made up of strongly acidic sulfated polysaccharide chains and has a mean molecular weight of 5 kDa (range 2–9 kDa). Dalteparin has a bioavailability of 87% when injected subcutaneously [97]. Numerous clinical trials compared subcutaneous dalteparin to UFH for the initial treatment of VTE (reviewed in [98]). Based on three metanalyses, dalteparin and other LMWHs are considered safe and as effective as conventional anticoagulant therapy in the treatment of acute VTE [99,100,101]. Dalteparin was shown to reduce the risk of recurrent VTE without significantly increasing the risk of major bleeding in patients with cancer and acute VTE. Other advantages over conventional anticoagulant therapy in this patient population include ease of administration at the time of invasive therapeutic procedures and thrombocytopenia induced by chemotherapy. Similarly to other LMWHs, dalteparin does not interact with other medications or poor diet, and there is no need for laboratory follow-up [98]. The extended use of dalteparin was evaluated in the population with cancer in the CLOT study, that randomized patients to VKA or extended-use dalteparin [94]. Symptomatic recurrent VTE at 6 months occurred in 27 (8%) patients treated with dalteparin for 6 months, and in 53 (15.8%) patients treated with dalteparin for 5–7 days followed by 6 months of treatment with VKA (RR reduction 52%; *p* = 0.002), whilst major bleeding occurred in 19 (6%) patients and 12 (4%) patients, in the dalteparin and in the dalteparin plus VKA arms, respectively, which was not statistically significant (*p* = 0.27). The study demonstrated that dalteparin was more effective, and as safe as conventional anticoagulant therapy in patients with cancer and acute VTE [94]. Other studies and real-world evidence showed similar results in terms of the efficacy and safety of dalteparin in populations with cancer [102,103]. The DALTECAN study was conducted to assess the safety of dalteparin beyond 6 months and within 12 months of treatment. It enrolled 334 patients of which 109 completed 12 months of therapy. The frequency of major bleeding was 10.2%. It was the most frequent in the first month and the least frequent in months 7–12 (3.6%, 1.1%, and 0.7% major bleeding per patient-month, respectively, for month 1, months 2–6 and 7–12) suggesting that the risk of bleeding was the highest in the first month of treatment [104].

#### 8.2.2. Tinzaparin

Tinzaparin has an average molecular weight of 6.5 kDa and is safe and efficacious for both short-term and long-term treatment of VTE in patients with cancer [105,106]. The efficacy of tinzaparin in the treatment of CAT was first investigated by the LITE study, which randomized 737 patients with acute DVT to receive tinzaparin or warfarin for 3 months. The results showed similar efficacy in both groups (VTE recurrence 4.9% and 5.7% in the tinzaparin and warfarin arms, respectively; absolute risk reduction (ARR)—0.8%, 95% CI, −4.1–2.4), whilst from the point of view of safety, it emerged that patients treated with tinzaparin developed a significantly lower number of bleeds (13% versus 19.8%, ARR—6.8%; *p* = 0.11; RR  =  0.66) [107]. Within this study, 200 patients with cancer were identified a priori and randomized separately. In this subpopulation, the rate of thromboembolic recurrence at 12 months follow-up in patients treated with tinzaparin was lower than in the group treated with dicoumarol by 7% and 13%, respectively (ARR—9.0; 95% CI, −21.7–0.7%) (Main-LITE cancer) [108]. This important finding was later confirmed in a larger multicenter trial, which randomized 900 patients with cancer and acute VTE to receive tinzaparin or warfarin for 6 months (CATCH study) [109]. The recurrence rate of VTE was lower in tinzaparin-treated patients, with a difference bordering on statistical significance (7.2% for tinzaparin versus 10.5% for warfarin; HR, 0.65; 95% CI, 0.41–1.03; *p* = 0.07). From a safety perspective, there was no difference in the incidence rate of major bleeds; however, significantly less clinically significant non-major bleeds occurred in the tinzaparin arm during the six months of treatment (49/449 patients for tinzaparin versus 69/451 patients for warfarin, HR 0.58; 95% CI, 0.40–0.84) [109].

A subsequent meta-analysis by Laporte et al, which included 24% of patients diagnosed with cancer among 1668 analyzed, demonstrated a non-significant reduction of 38% (RR: 0.62: *p* = 0.21) in the risk of VTE at 3–6 months, increased to 59% at 12 months, bordering on statistical significance in this patient population (RR: 0.41: *p* = 0.08) [110]. Another meta-analysis, which analyzed three trials that compared tinzaparin and dicoumarols (LITE, CATCH, and subpopulation study by Romera et al. [111]) reached similar conclusions showing a reduction in the recurrence rate of VTE (RR 0.67, 95% CI, 0.46–0.99) without a difference in the number of major bleeds [112].

The TiCAT study, instead, analyzed the efficacy and safety of treatment with tinzaparin as a secondary prophylaxis after a 6-month-long initial treatment period. The rate of clinically relevant bleeds was found to be 0.9% of patients in the first 6 months and 0.6% of patients in the period between 7 and 12 months, while the recurrence rate of VTE was 4.5% during the first period and 1.1% in the following 6 months [113]. Also, the post hoc analysis of data from the TROPIQUE study confirmed the favorable benefit–risk ratio of tinzaparin for the long-term treatment of CAT [114].

Long-term therapy with tinzaparin was also tested as a home treatment in the Home-LITE study [115]. In this study, 478 patients were randomized to receive tinzaparin or warfarin for 1 year. The study reported a similar risk of thromboembolic recurrence and major bleeding but with a clear patient preference for parenteral drugs. When asked to express a degree of satisfaction with the therapy received, the majority of patients assigned more favorable evaluations to the injectable therapy (*p* = 0.024) [115]. It has to be noted that this study precedes the era of the widespread use of DOACs.

Tinzaparin has a favorable pharmacokinetic profile in patients with renal insufficiency. A recent meta-analysis reported that patients with renal insufficiency and cancer treated with tinzaparin had a significant reduction in major bleeding events when compared to those treated with warfarin [89], demonstrating that tinzaparin is safe in a population at high risk of bleeding. Furthermore, when compared with enoxaparin, tinzaparin was shown not to accumulate in a statistically significant way [116].

In a real-world setting, tinzaparin was administered to 407 patients with cancer, and CAT was included in the Greek study of Management of Thrombosis. A dose of tinzaparin of 8000–12,000 Anti-Xa IU was found to potentially be more effective than the prophylactic dose of ≤4500 Anti-Xa IU, both administered once daily. Such an intermediate dose, along with the administration of erythropoietin, was received more often by patients with a body mass index >30 kg/m^2^, those with metastases, and those who were ex-smokers, and was associated with no safety issues [117].

The validity of the Ottawa score was assessed in 409 patients who were given prophylaxis with tinzaparin for 6 months. In this study, the application of the Ottawa score did not predict the recurrence of VTE; the overall rate of recurrent VTE was 7.3% (CI, 4.9–11.1) [118].

#### 8.2.3. Enoxaparin

Enoxaparin is an LMWH that was first approved for medical use in 1993. It has a molecular weight of 4 to 5 kDa and a bioavailability of 91% when administered subcutaneously. Enoxaparin is eliminated mainly into the urine thus requiring a dose adjustment in patients with a creatinine clearance < 30 mL/minute [119].

The efficacy and safety of enoxaparin versus VKAs for long-term therapy of VTE in patients with cancer were demonstrated in two albeit relatively small RCTs: CANTHANOX which enrolled 146 patients and ONCENOX which enrolled 102 patients. Both studies included patients with solid or hematological tumors and VTE [120,121]. In the real-world setting, the effectiveness and safety of enoxaparin versus dalteparin and tinzaparin were recently compared in patients with cancer in the RIETECAT study. The study included 4451 patients treated with enoxaparin, 754 with dalteparin, and 171 with tinzaparin, all at full doses. A limited difference was observed in VTE recurrences (2.0% versus 2.5%) and mortality rate (19% versus 17%) between the enoxaparin and dalteparin or tinzaparin groups. Compared to the tinzaparin and dalteparin groups, there was an increase in the major bleeding rate in the enoxaparin group (3.1% vs. 1.9%) [122].

### 8.3. Fondaparinux

The comprehension of the mechanism of the anticoagulant action of heparin resulted in the creation of synthetic mimetics, which seemed an attractive and less heterogeneous alternative to natural heparins. In 2001, fondaparinux, a synthetic and selective inhibitor of factor Xa, was registered in the United States and Europe as a new antithrombotic drug. It takes more than 50 steps to synthesize fondaparinux and the overall yield of the process is 0.1% [123]. Fondaparinux has a molecular weight of 1.728 kDa and is a selective and reversible inhibitor of factor Xa dependent on binding to antithrombin to elicit its activity.

Unlike heparin and heparan sulfate, there is little binding and inhibition of factor IIa or other circulating proteins by fondaparinux. Moreover, fondaparinux has been successfully used with other therapeutics [124].

Because of the half-life of 17 h, the bleeding risks of fondaparinux should be carefully assessed. The choice of long-acting anticoagulants requires adequate reversibility by an antidote in cases of bleeding. There is limited experience with the reversal of fondaparinux in the case of bleeding [125].

An assessment of the efficacy, safety, and overall survival when treated with fondaparinux compared to standard initial treatment with LMWH in patients with cancer and VTE was completed in two post hoc analyses from two RCTs. Initially, 237 patients with DVT and 240 patients with PE were treated, respectively, with fondaparinux or enoxaparin and with fondaparinux or UFH. All patients went on to receive vitamin K antagonists. In patients with DVT, the 3-month recurrence rate was 5.4% and 12.7% (absolute difference −7.3%, 95% CI, 0.1–14.5) in the enoxaparin and fondaparinux recipients, respectively. In patients with PE, the recurrence rate was 8.9 % and 17.2% (absolute difference −8.3, 95% CI, −16.7–0.1) in patients treated with fondaparinux and those with UFH, respectively. Overall, no differences in bleeding frequency and overall survival were observed [126].

Similar results regarding the safety and efficacy of fondaparinux in comparison with enoxaparin were obtained in a meta-analysis of four multicenter, randomized, double-blind trials [127]. The incidence of VTE was halved from 13.7% to 6.8% with LMWH versus fondaparinux, respectively [127]. FDA approved fondaparinux in the initial treatment of VTE [128]. The placement of a vena cava filter in addition to anticoagulation with fondaparinux sodium provided no advantage in terms of safety, recurrent thrombosis, recurrent pulmonary embolism, or survival in a prospective randomized trial evaluating anticoagulation plus a vena cava filter in 66 patients with cancer and DVT (86%) and/or PE (55%) [129]. More studies have to be conducted to fully evaluate the safety and efficacy of this agent in patients with cancer.

### 8.4. Anticancer Effect

Heparins, including UFH, LMWHs, and heparin derivatives, have been reported to have beneficial effects on cancer survival. Heparin affects the fundamental molecular steps involved in cancer progression, i.e., proliferation, angiogenesis, cell migration, adhesion, and invasion. Multiple mechanisms are responsible for the anticancer properties of heparin and derivatives, the main being heparinase inhibition, and blocking the CXCL12-CXCR4 axis [130].

In vitro studies demonstrated that tinzaparin has anti-angiogenic and anti-metastatic properties [105]. Out of three LMWHs tested, tinzaparin showed increased selectin inhibition. Experimental models showed metastasis attenuation upon treatment with UFH and tinzaparin, at clinically relevant anticoagulation levels. This may be due to the fact that tinzaparin contains high molecular weight fragments, which are not present in other LMWHs, responsible for selectin inhibitory activity [131]. Preincubation of the chemoresistant A2780cis cell line led to sensitization to cisplatin [132] possibly through the Wnt signaling pathway [133]. Karamouzis et al. demonstrated in a recent meta-analysis that the administration of tinzaparin in patients diagnosed with advanced pancreatic cancer increased the disease-free period by 39.5% compared to the population that did not receive the drug [134]. Enoxaparin was found to inhibit the CXCL12-driven proliferation, adhesion, and colony formation of human colon cancer HCT-116 cell line and suppressed the formation of metastases in Balb/C mice [135]. The anti-cancer mechanisms of heparin and its derivatives, some of which have no anticoagulative properties, were reviewed by Ma et al. [130].

## 9. Conclusions

CAT is associated with significant morbidity and mortality and its treatment continues to be a challenge. It is important to both identify patients at high risk of VTE who may benefit from primary prophylaxis and to treat the thrombotic events in the most appropriate manner. To do this, different therapeutic strategies are now available: there is evidence for the efficacy and safety of both injectable anticoagulants extensively reviewed here and of DOACs. Injectable anticoagulation, and in particular LMWHs, may be preferred according to the site of cancer, drug–drug interactions, individual bleeding risk, and the presence of renal and/or hepatic insufficiency. Amongst LMWHs, the usefulness of dalteparin, tinzaparin, and to a lesser extent enoxaparin, has been extensively confirmed in patients with malignancies and all three are approved for the acute and extended treatment of VTE in cancer.

Given the evolving trends in the use frequency of different anticoagulant classes [136], the advantages and disadvantages of DOACs must briefly be mentioned here. This new class, introduced in 2010, has a rapid onset of action, requires no monitoring, is conveniently administered by mouth at fixed dosages, and has predictable pharmacokinetics and pharmacodynamics. Moreover, DOACs have a wide therapeutic window and reversal agents are available. The disadvantages enumerate still less extensive clinical experience than that of LMWHs, and decreased efficacy in patients who vomit or who have GI system alterations leading to altered absorption. In patients at a high risk of bleeding, DOACs should also be avoided, as some studies showed a higher incidence of bleeding. DOACs may also interfere with the action of specific anticancer therapies. Together, DOACs have probably become a replacement for VKA and are a valid alternative option to LMWH in most cases [137].

As the prevalence of cancer continues to increase because of the aging population and prolonged survival of patients with advanced malignancies, the burden of CAT will remain a serious clinical reality.

## Figures and Tables

**Table 1:** Comparison of the characteristics of the major anticoagulant classes.

	Vitamin K Antagonists	Low Molecular Weight Heparins	Direct Oral Anticoagulants
Route of intake	Oral	Subcutaneous injection	Oral
Problems with oral intake in cancer	Yes	No	Yes
Problems with absorption in cancer	Yes	No	Yes
Renal clearance	No	Yes (except tinzaparin)	Yes
Food interactions	Yes	No	Yes
Influence of fasted/fed status	No	No	Yes *
Pharmacokinetic drug–drug interactions	Yes, with chemotherapeutics	No	Yes
Need to monitor	Yes	Not routine	No

* Rivaroxaban should be taken with food. Prolonged fasting in patients on DOAC treatment may affect drug absorption leading to ineffective therapy [60].

**Table 2:** Comparison of the characteristics of the major injectable anticoagulants.

	Unfractionated Heparin	Low Molecular Weight Heparins	Fondaparinux
Origin	Natural compound isolated from animal liver	Depolymerization of heparin	Synthetic compound
Molecular weight	15–19 kDa	3–6.5 kDa	1.7 kDa
Target of inhibition	Factors Xa and IIa, a weak anti-platelet effect	2–4 times more efficient at inhibiting factor Xa than IIa	Factor Xa
Half-life	~1 h	3–6 h *	17–21 h *
Metabolism/excretion	Reticuloendothelial and renal	10–40% renal	Renal
Antidote	Protamine	Protamine (partial reversal **)	None
Interaction with platelets	Strong	Weak	None
Risk of HIT	Strong	Weak	None

* If normal renal function, for fondaparinux also age dependent; ** reversal efficiency rates for the three LMWHs described in this review are 74% for dalteparin, 85.7% for tinzaparin, and 54.2% for enoxaparin [87]; HIT, heparin-induced thrombocytopenia.

**Table 3:** Dosing of unfractionated heparin, of low molecular weight heparins, and of fondaparinux according to ESMO guidelines for primary prophylaxis and treatment of cancer-associated thrombosis [7].

Anticoagulant	Clinical Setting		
	Inpatients	Patients Undergoing Surgery	Outpatients
**Primary Prophylaxis**			
Unfractionated heparin	5000 IU every 8 h	5000 IU 2–4 h before surgery and every 8 h thereafter	NA
Dalteparin	5000 anti-Xa IU	5000 anti-XaIU 12 h before surgery and 5000 anti-Xa IU once daily thereafter	5000 anti-Xa IU once daily
Enoxaparin	4000 anti-Xa IU	4000 anti-Xa IU 12 h before surgery and 4000 anti-Xa IU once daily thereafter	4000 anti-Xa IU once daily
Tinzaparin	4500 anti-Xa IU	4500 anti-Xa IU once daily starting 12 h post-surgery	4500 anti-Xa IU once daily
Fondaparinux	2.5 mg once daily	2.5 mg once daily starting 6–8 h post-surgery	No data
**Initial treatment**			
Unfractionated heparin	80 IU/kg intravenous bolus, next 18 IU/kg/h intravenously; dose adjusted based on aPTT		
Dalteparin	100 anti-Xa IU/kg every 12 h, or 200 anti-Xa IU/kg once daily up to day 30		
Enoxaparin	100 anti-Xa IU/kg every 12 h, or 150 anti-Xa IU/kg once daily		
Tinzaparin	175 anti-Xa IU/kg once daily		
Fondaparinux	Not listed in this setting		
**Extended treatment**			
Unfractionated heparin	Not completed		
Dalteparin	150 anti-Xa IU/kg once daily beyond day 30		
Enoxaparin	100 anti-Xa IU/kg every 12 h, or 150 anti-Xa IU/kg once daily		
Tinzaparin	175 anti-Xa IU/kg once daily		
Fondaparinux	Not listed in this setting		

aPTT, activated partial prothrombin time; IU, international unit.

## Data Availability

Not applicable.

## Supplementary Materials

The following supporting information can be downloaded at: https://www.mdpi.com/article/10.3390/cancers15184640/s1, Table S1: Summary of clinical trials at the basis of the current international guidelines for the management of cancer-associated thrombosis.
