# Supplementary material for: The Role of Injectables in the Treatment and Prevention of Cancer-Associated Thrombosis
Source: Cancers (Basel). 2023 Sep 20;15(18):4640. doi: 10.3390/cancers15184640 (PMC10526875; doi:10.3390/cancers15184640)
Supplement: Supplementary file 1 [file cancers-15-04640-s001.zip › cancers-2498548-supplementary.pdf]

Supplementary Table S1. Summary of clinical trials at the basis of the current international guidelines for the management of cancer-associated thrombosis.

Study	Number of patients	Anticoagulant used	Comparator	Primary outcomes		VTE events (%)	HR (95% IC) for recurrence of VTE	Major bleeding (%)	HR (95% IC) for major bleeding
Injectable anticoagulants versus VKA									
**CLOT**	676	Dalteparin	VKA	Recurrence of VTE		7 vs 15	0.48 (0.30–0.77)	6 vs 4	NA
**Main-LITE**	737	Tinzaparin	Usual care (UFH and VKA)	Effectiveness: Recurrence of VTE	Safety: All bleeding	4.9 vs 5.7 at 3 months	AD: -0.8 (-4.1-2.4)	13 vs 19.8	NA
**Home-LITE**	480	Tinzaparin	Usual care (tinzaparin plus VKA)	Recurrence of VTE		3.3	NA	No difference	NA
**CANTHANOX**	146	Enoxaparin	VKA	Combined outcome (recurrent VTE or major bleeding)		10.5 vs 21.1	RR: 2.02 (0.88-4.65)	7 vs 16	NA
**ONCENOX**	122	Enoxaparin (low and high dose)	VKA	Recurrence of VTE		6.3 (HD) vs 6.9 (LD) vs 10	NA	11.1 (HD) vs 6.5 (LD) vs 2.9	NA
**RIETECAT**	4451	Enoxaparin	Tinzaparin or dalterapin	Recurrence of VTE		2 vs 2.5	0.81 (0.48-1.38)	3.1 vs 1.9	1.4 (0.8-2.46)
**CATCH**	900	Tinzaparin	VKA	Effectiveness: Recurrence of VTE or death	Safety: All bleeding	6.9 vs 10	0.65 (0.41–1.03)	2.7 vs 2.4	0.89 (0.4-1.99)
DOACs versus LMWHs									
**HOKUSAI-VTE CANCER**	1050	Edoxaban	Dalteparin	Recurrence of VTE + major bleeding		7.9 vs 11.3	0.71 (0.48–1.06)	6.9 vs 4	1.77 (1.03–3.04)
**SELECT-D**	406	Rivaroxaban	Dalteparin	Recurrence of VTE		4 vs 11	0.43 (0.19–0.99)	6 vs 4	1.83 (0.68–4.96)
**CASTA-DIVA**	158	Rivaroxaban	Dalteparin	Major bleeding		6.4 vs 10.1	0.75 (0.21–2.66)	1.4 vs 3.7	0.36 (0.04–3.43)

Study	Number of patients	Anticoagulant used	Comparator	Primary outcomes		VTE events (%)	HR (95% IC) for recurrence of VTE	Major bleeding (%)	HR (95% IC) for major bleeding
**ADAM-VTE**	300	Apixaban	Dalteparin	Effectiveness: Recurrence of VTE	Safety: Major bleeding	0.7 vs 6.3	0.099 (0.013–0.780)	0 vs 1.4	Not computable
**CARAVAGGIO**	1170	Apixaban	Dalteparin	Effectiveness: Recurrence of VTE + worsening of vascular or venous obstruction.	Safety: Major bleeding	5.6 vs 7.9	0.63 (0.37–1.07)	3.8 vs 4	0.82 (0.40–1.69)
**CANVAS**	671	DOACs	LMWH	Effectiveness: Recurrence of VTE	Safety: Major bleeding	6.1 vs 8.8	Not reported	5.2 vs 5.6	Not reported

AD: absolute difference; DOAC: direct oral anticoagulant; HR: hazard ratio; IC: confidence interval; LMWH: low molecular weight heparin; RR: relative risk; VKA: vitamin K antagonists; vs: versus; VTE: venous thromboembolism.
